# THE POWER OF SHARED EXPERIENCE: VALIDATION AS A CATALYST FOR MEANING MAKING AMONG ADRD CAREGIVERS

**DOI:** 10.1093/geroni/igae098.3749

**Published:** 2024-12-31

**Authors:** Jacquelyn Benson, Abigail Haber, Karla Washington, Abigail Rolbiecki

**Affiliations:** Washington University in St. Louis, St Louis, Missouri, United States; Albert Einstein College, New York, New York, United States; Washington University in St. Louis, St. Louis, Missouri, United States; University of Colorado Anschutz, Aurora, Colorado, United States

## Abstract

Caregiver Speaks is a professionally facilitated online group intervention designed to support hospice family caregivers of patients with dementia by using photos as a tool for expressing and processing their caregiving experiences. The structured intervention enables ADRD caregivers to articulate their feelings, challenges, and experiences, with the primary goals of reducing caregiver distress, alleviating grief, and enhancing overall well-being. The intervention emphasizes helping ADRD caregivers find personal significance in their roles through photo-elicited communication, fostering meaningful emotional expression and reflection. The primary objective of the current study was to evaluate ADRD caregivers’ descriptions of their experiences in the Caregiver Speaks intervention to determine how their interactions in the group facilitated meaning making. Forty-one (41) exit interviews were analyzed using a constant comparative method involving an iterative process of deductive and inductive coding. Results indicated that caregivers’ interactions with others in the group primarily evoked a sense of solidarity, although some outliers—particularly those with strained relationships with their care recipients—reported feelings of alienation or disconnection. While direct dialogue between group members was infrequent, caregivers found reconciliation in their caregiving experiences and their care recipient’s illness by seeing their own experiences reflected in the photos and stories shared by others. The indirect validation provided by fellow participants was particularly impactful for many caregivers, who often lacked connections with other dementia caregivers. These findings underscore the critical roles of shared lived experience and validation in the meaning-making process.

